# Uptake of health economic evaluations alongside clinical trials in Australia: an observational study

**DOI:** 10.1186/s13063-024-08562-3

**Published:** 2024-10-22

**Authors:** Alayna Carrandi, Cynthia Wells, Rachael L. Morton, Richard Norman, Helen Skouteris, Amy Grove, Alisa M. Higgins

**Affiliations:** 1 Australian and New Zealand Intensive Care Research Centre (ANZIC-RC), Department of Epidemiology and Preventive Medicine, School of Public Health and Preventive Medicine, Monash University, Melbourne, VIC Australia; 2 Clinical Trials Centre, Faculty of Medicine and Health, National Health and Medical Research Council (NHMRC, University of Sydney, Camperdown, NSW Australia; 3 Faculty of Health Sciences, School of Population Health, Curtin University, Perth, WA Australia; 4 Health and Social Care Unit, School of Public Health and Preventive Medicine, Monash University, Melbourne, VIC Australia; 5 Warwick Medical School, Warwick University, Coventry, UK

**Keywords:** Cost-benefit analysis, Cost-effectiveness analysis, Evidence-based medicine, Randomized controlled trials

## Abstract

**Background:**

Australia’s clinical trials sector is highly productive with continued sector investment needed to enhance research impact. Generating economic evidence alongside trials has the potential to facilitate the implementation of trial results into practice. Ascertaining the use of health economic evaluations alongside clinical trials can assist in determining whether clinical trials fully realize and operationalize their potential to change policy and practice. The aims of this study were to ascertain the uptake of health economic evaluations alongside Australian-led clinical trials and explore associations between uptake and trial characteristics.

**Methods:**

This observational study comprised a descriptive analysis of clinical trials registries, a cross-sectional survey of Australian Clinical Trials Alliance (ACTA) networks, and a subgroup analysis of completed acute care trials. Descriptive analyses of trial registrations were conducted, with logistic regressions used to identify predictors of proposing and subsequently publishing a health economic evaluation alongside acute care trials.

**Results:**

Few randomized Australian-led clinical trials (11% of 9251) and ACTA network trials (43% of 227) proposed a health economic evaluation. In the subgroup analysis, 22% of the 324 acute care trials and 53% of the 38 ACTA network acute care trials proposed a health economic evaluation. Acute care trials funded by government bodies were significantly more likely to propose and publish a health economic evaluation than those funded by hospitals, universities, and other funders, after adjusting for phase, registration year, primary sponsor type, and comparator.

**Conclusions:**

Current uptake of health economic evaluations alongside Australian-led clinical trials is low, with uptake higher among the subset of ACTA network trials. This is despite economic evidence playing an increasingly prominent role in health system management, as well as rising health expenditure, limited budgets, and competing demands. There is significant opportunity to embed health economic evaluations alongside clinical trials, particularly phase 3 trials, to increase research outputs and optimize research translation. Investing in clinical trial networks that support funding for a health economist or a health economic evaluation may be an effective strategy to increase the uptake of health economic evaluations alongside trials.

**Supplementary Information:**

The online version contains supplementary material available at 10.1186/s13063-024-08562-3.

## Background

Clinical trials are designed to evaluate the safety, efficacy, or effectiveness of one or more health care interventions. Determining that an intervention is effective, however, is no longer sufficient to guarantee its implementation into clinical practice [1]. This is due to rapidly increasing healthcare costs and demand for resources outpacing supply [2]. A health economic evaluation, therefore, may be undertaken alongside a clinical trial to inform resource allocation decisions and ensure clinical practice reflects that which is most effective and efficient.

Australia has demonstrated above-average clinical trial productivity (number of trials per capita) compared with other OECD countries [3]. Australia’s industry and government sectors have continued to prioritize investment in the clinical trial landscape to enhance research impact, i.e., increase research outputs and optimize research translation [4, 5]. Investment in Australian clinical trial networks, comprising investigator-initiated clinical trials, has been one effective strategy driving research impact. The value of investigator-initiated clinical trials is evidenced by high implementation rates of trial evidence into practice [6]. Implementation rates are also influenced by an understanding of the investment required to implement an intervention and the value of its implementation in practice [7]. The Australian government has recognized the importance of economic evidence to support resource allocation decisions and the need to expand health economic capacity in Australia [8]. Generating economic evidence alongside clinical trials can further improve and accelerate the implementation of trial results into practice.

Despite the established value of health economic evaluations alongside clinical trials [9], it is currently unknown how often these evaluations are proposed, completed, and published. Ascertaining the uptake of health economic evaluations alongside clinical trials is important to document trends in the field and identify potential evidence and knowledge gaps. Documenting trends in the field can assist in determining whether clinical trials fully realize and operationalize their potential to change policy and practice using health economic evaluations. The aims of this study were to ascertain the uptake of health economic evaluations alongside Australian-led clinical trials, to explore the associations between trial characteristics and uptake of health economic evaluations, and to determine whether uptake of health economic evaluations differs between clinical trial networks and all trials.

## Methods

This observational study design comprised three quantitative components: (1) a descriptive analysis of health economic evaluations within clinical trials registries; (2) a cross-sectional survey of Australian clinical trial networks; and (3) a subgroup analysis of therapeutic areas with high uptake of health economic evaluations. The Australian New Zealand Clinical Trials Registry (ANZCTR) [10] and ClinicalTrials.gov [11] registries were systematically searched to identify all Australian-led interventional clinical trials registered from January 2005 to June 2023. Australian clinical trial networks who are members of the Australian Clinical Trials Alliance (ACTA) were surveyed to ascertain the trials endorsed or run by networks. These data were combined and descriptively analyzed to explore associations between trial characteristics and proposing a health economic evaluation. A subgroup analysis was then used to further explore the predictors of proposing and publishing a health economic evaluation among therapeutic areas. We undertook a subgroup analysis to feasibly validate the results found in the registries, as trial registrations are not always regularly updated [3]. The acute care subgroup, comprising emergency medicine, critical care, surgery, and injury, was chosen based on the therapeutic areas’ number of completed trials with a health economic evaluation. Research ethics committee approval was not required as this study is a review of publicly available data. Reporting followed the Strengthening the Reporting of Observational Studies in Epidemiology (STROBE) statement (Additional file 1) [12].

### Analysis of clinical trials registries

All trial records from the public ANZCTR website with Australia listed as at least one of the countries of recruitment were downloaded on 16 June 2023 [10]. Trials with Australian recruitment locations and registered since 2005 (ANZCTR inception) were downloaded from the public ClinicalTrials.gov website on 20 July 2023 [11]. Trial registrations were then uploaded into the R software. Registrations were filtered using R code to capture only Australian-led randomized phase 3 and phase 4 interventional trials. The code used to filter trial registrations is publicly available ([13]; Additional file 2).

Australian-led trials were defined as trials where the institution of the lead investigator or the sponsor lead was based in Australia. Multinational trials with Australian sites where the institution of the lead investigator or sponsor lead was not in Australia were excluded. The location of the lead investigator’s or sponsor lead’s institution was available on ANZCTR registrations. Trials registered with ClinicalTrials.gov were screened by one reviewer (AC) to ascertain whether the location of the lead investigator’s institution or sponsor lead was Australia. Interventional trials regardless of recruitment status were included. Trial registrations were included where the trial phase was recorded as “not applicable” (N/a) or “phase 2/3.” Phase N/a is used to describe trials of devices or behavioral interventions (i.e., non-drug trials) without defined phases. Phase 1, phase 2, and observational trials were excluded. Duplicates were removed, where trials were registered with both ANZCTR and ClinicalTrials.gov.

The ANZCTR registration form allows sponsor leads and investigators to categorize their trial into various therapeutic areas. Search terms related to established ANZCTR therapeutic areas were used to categorize trials registered only with ClinicalTrials.gov. An additional category, critical care, was created by searching for related terms listed in the title, intervention, and health condition fields of ANZCTR and ClinicalTrials.gov registrations. Trials could be categorized into more than one therapeutic area. Search terms, such as “cost,” “quality-adjusted life-year,” “cost-effectiveness,” and “incremental net benefit,” were used to identify trials with a health economic evaluation. Trials were categorized as having a health economic evaluation if they included the aforementioned terms in the title, summary, outcomes, or statistical analysis fields of registrations.

### Survey of clinical trial networks

The Australian Clinical Trials Alliance (ACTA) is the national peak body for supporting and representing networks of clinician-researchers conducting investigator-initiated clinical trials in Australia. ACTA comprises approximately 47 clinical trials networks. ACTA network representatives were invited via email to provide a list of their networks’ ongoing (i.e., have commenced recruitment) and completed trials. This included any trials that the network has endorsed or run since 2005 (ANZCTR inception). If the network did not provide a list of their ongoing and completed trials, the project team attempted to retrieve a list of trials from the network website or annual reports.

### Subgroup analysis

A subgroup analysis was conducted for acute care trials, comprising emergency medicine, critical care, surgery, and injury. This subgroup was chosen because there was good representation of completed trials with a health economic evaluation (*n* = 52/433 completed trials; 12%). This subgroup was also well-represented among ACTA networks (*n* = 39/227 ACTA network trials; 17%).

All acute care trials where the trial registration’s recruitment status was “complete” were extracted into an excel spreadsheet (Additional file 3). Additional trial information was retrieved by one reviewer (AC) from the trial registration, study protocol, or publications. Associated publications, including the primary publication and health economic evaluation, were retrieved from the trial registration, Google, and academic databases. Only trials with primary results published were included. Pilot trials were excluded where the trial phase had been recorded as “N/a.” Pilot trials do not typically incorporate health economic evaluations due to small sample sizes and the focus on feasibility and acceptability of the intervention and trial design [14]. A trial had a proposed health economic evaluation if the registration, protocol, or statistical analysis plan reported cost-effectiveness, cost-minimization, or cost-utility in the analysis or outcome measures. Trials that only collected health resource utilization data without reporting an analysis of cost and consequences were not considered to have a proposed health economic evaluation. The health economic evaluation was considered “ongoing” if the trial had proposed a health economic evaluation, the primary results had been published in the last 5 years, but the health economic evaluation had not yet been published. The results section of the primary publication was reviewed to determine if the primary outcome result was statistically significant.

#### Data analysis

A descriptive analysis of all Australian-led clinical trials registered with ANZCTR or ClinicalTrials.gov and the subgroup of completed acute care trials was conducted to determine the characteristics of trials with and without a proposed health economic evaluation. Univariate analyses were used to explore associations between trial characteristics and whether a trial proposed and/or published a health economic evaluation. For the subgroup analysis of completed acute care trials, logistic regression analysis was used to explore variables that were independently associated with proposing and/or publishing a health economic evaluation. A *p* value < 0.05 was considered statistically significant.

## Results

### All Australian-led clinical trials

**Fig. 1:**
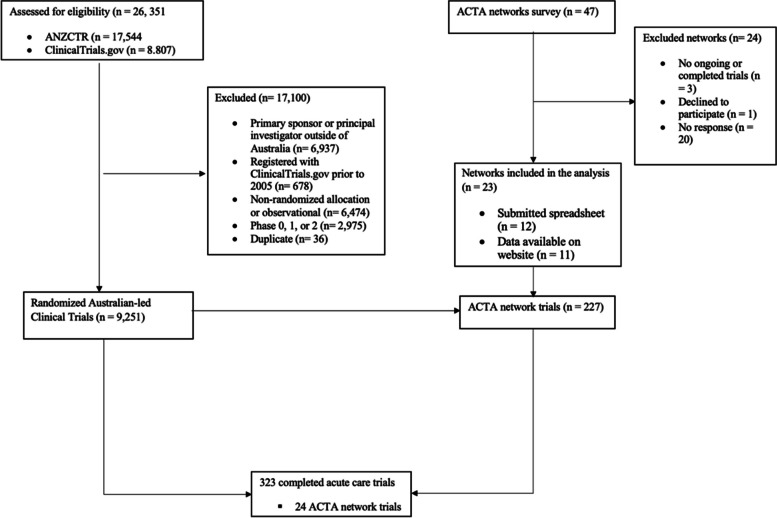
Flow diagram

Of the 26,351 trials assessed for eligibility, 9251 were Australian-led interventional randomized trials (Fig. 1), of which 2% (*n* = 227) were ACTA network trials (Additional file 4).

**Fig. 2:**
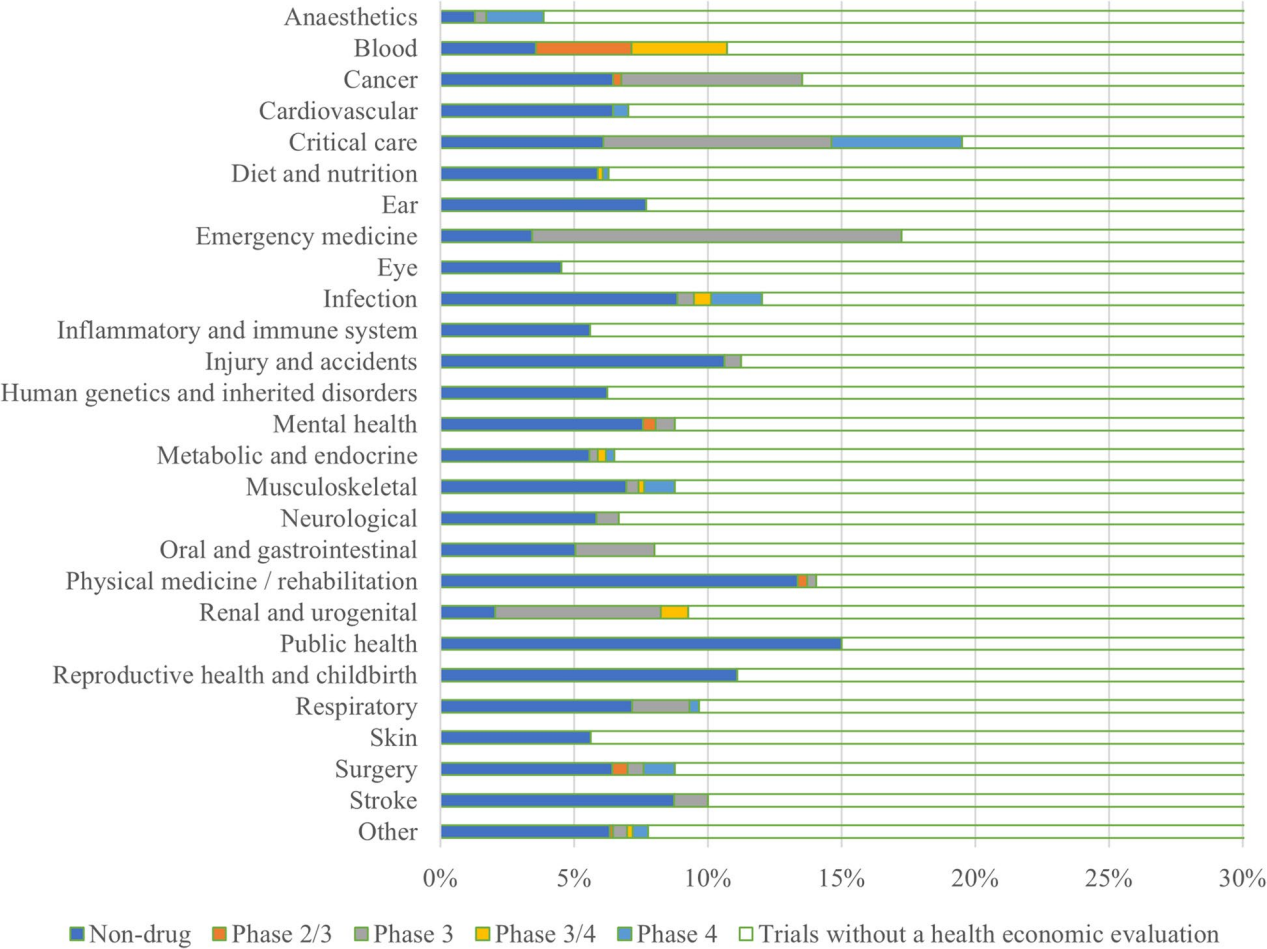
Proportions of completed trials with a health economic evaluation, by therapeutic area

Most (*n* = 8217; 89%) Australian-led trials did not propose a health economic evaluation (Additional file 4), and approximately 43% (*n* = 98) of ACTA network trials proposed a health economic evaluation. Among different therapeutic areas, emergency medicine and critical care had the largest proportion of completed trials with a proposed health economic evaluation (17% and 20% respectively) (Fig. 2). The proportion of all trials with a proposed health economic evaluation increased over time (9% in 2005–2011 to 18% in 2019–2023).

### Subgroup analysis of completed acute care trials

Of the 324 completed acute care trials identified, 16% (*n* = 52) proposed a health economic evaluation (Table 1; Additional file 5), and over half (*n* = 20/38; 53%) of ACTA network acute care trials proposed a health economic evaluation.

**Table 1:** Characteristics of completed acute care trials with a proposed health economic evaluation, including emergency medicine, critical care, surgery, and injury trials

**Descriptive characteristic**	**Total**		**Trials with a proposed health economic evaluation**	
	*n* = 324		*n* = 52	
Phase	*n*	%	*n*	%
Not applicable (non-drug trials)	222	68.5	32	61.5
Phase 2/3	8	2.5	2	3.9
Phase 3	39	12.0	14	26.9
Phase 3/4	6	1.9	0	0.0
Phase 4	49	15.1	4	7.7
Registration date	Median	IQR	Median	IQR
Year	2014	2010–2017	2014	2011–2016
ACTA network trial	*n*	%	*n*	%
Yes	38	11.7	20	38.5
Primary funding source^a^	*n*	%	*n*	%
Government body	84	25.9	33	63.5
Hospital	75	23.2	5	9.6
University	19	5.9	1	1.9
Commercial sector/industry	23	7.1	4	7.7
Charities/societies/foundations	50	15.4	5	9.6
Other collaborative groups	17	5.3	3	0.9
Self-funded/unfunded	48	14.8	1	1.9
Other	8	2.5	0	0.0
Primary sponsor type	*n*	%	*n*	%
Government body	5	1.5	1	1.9
Hospital	117	36.1	13	25.0
University	66	20.4	17	32.7
Commercial sector/industry	8	2.5	2	3.9
Charities/societies/foundations	10	3.1	2	3.9
Other collaborative groups	18	5.6	5	9.6
Individual	90	27.8	8	15.4
Other	7	2.2	1	1.9
None	3	0.9	0	0.0
Comparator	*n*	%	*n*	%
Standard care	141	43.5	31	59.6
Wait-list control	8	2.5	0	0.0
Placebo	31	9.6	1	1.9
Cross-over trial	5	1.5	0	0.0
Other	139	42.9	20	38.5

*ACTA* Australian Clinical Trials Alliance, *IQR* Interquartile range

^a^Significant (*p* < 0.05) independent association with proposing and publishing a health economic evaluation, accounting for phase, registration year, primary sponsor type, and comparator

Most acute care trials with a proposed health economic evaluation were non-drug (*n* = 32; 62%) or phase 3 trials (*n* = 14; 27%). The most common primary funding source among trials with a proposed health economic evaluation was government bodies (*n* = 33; 63%), and the most common primary sponsor type was universities (*n* = 17; 33%). Conversely, trials without a proposed health economic were typically funded (*n* = 70; 26%) and sponsored (*n* = 104; 38%) by hospitals. Standard care was the most common comparator among trials with a proposed health economic evaluation (*n* = 31; 60%). The primary funding source was significantly (*p* < 0.05) associated with proposing and publishing a health economic evaluation, after accounting for registration year, phase, primary sponsor type, and comparator (Table 1).

Among the 52 completed acute care trials with a proposed health economic evaluation, many health economic evaluations (*n* = 25; 48%) were considered ongoing, i.e., the primary results from the trial have been published within the last 5 years. Among the remaining 27 completed acute care trials with a proposed health economic evaluation, 14 published a health economic evaluation. The most common economic methodology used was cost-utility analysis (*n* = 11; 79%). The median time between primary publication and health economic evaluation publication was 2 years (interquartile range: 2–3). All health economic evaluations were published independently from the primary publication. Most (*n* = 12; 86%) published health economic evaluations used only trial-based data [15–26], except for two cost-utility analyses of implementation strategies that modeled cost-effectiveness inputs [27, 28]. Most trials that proposed but did not publish a health economic evaluation (*n* = 8; 62%) reported non-significant findings for the trial’s primary outcome.

## Discussion

Of the 9251 randomized Australian-led clinical trials identified across ANZCTR and ClinicalTrials.gov, 11% proposed a health economic evaluation. Of the 227 trials run by ACTA networks, 43% proposed a health economic evaluation, which is nearly four times the proportion (11%) of all trials with a health economic evaluation. We identified 324 completed acute care trials (inclusive of emergency medicine, critical care, injury, and surgery trials), of which 38 were ACTA network trials. Approximately 16% of completed acute care trials proposed a health economic evaluation, whereas 53% of ACTA network acute care trials proposed a health economic evaluation. Acute care trials funded by government bodies were significantly more likely to propose and publish a health economic evaluation than trials funded by other sources, after adjusting for phase, registration year, primary sponsor type, and comparator.

The uptake of health economic evaluations alongside Australian-led trials is rising over time (9% in 2005–2011 to 18% in 2019–2023), in line with the increasingly prominent role of economic evidence in health system management. However, most (89%) Australia-led interventional randomized trials did not include a health economic evaluation. Although phase 3 trials comprised the largest proportion (13%) of non-drug trials with a health economic evaluation, this remains an area for growth. Phase 3 trials represent an opportune time to generate economic evidence for an intervention prior to being introduced to market. Even clinical trials with non-significant differences in effectiveness between interventions would benefit from a health economic evaluation because findings may illuminate an opportunity to reduce costs without impacting patient outcomes. Not only can health economic evaluations establish evidence for new cost-effective interventions, but they can also inform disinvestment from costly interventions that do not realize health gains.

Increasing capacity to conduct health economic evaluations alongside clinical trials can ensure the investment in and implementation of evidence-based interventions that maximize value. However, we do not yet know the extent to which trial results translate into routine practice. Implementation strategies, outcomes, and costs are rarely measured in clinical trials [7], which limits our understanding of how to efficiently implement clinical trial findings into practice. We identified two acute care trials assessing the cost-effectiveness of implementation strategies [27, 28]. Reporting implementation strategies used in clinical trials and aligning these strategies with implementation outcomes and costs may expedite the research translation process by establishing the value of implementing interventions in a local context. This may be especially important in contexts, such as acute care, where spending is high and creating efficiencies can have a substantial impact.

Global health systems, including the UK [29] and Australia [30], are increasingly recognizing the value of investing in clinical trial research and building local capacity to conduct clinical trials. Government bodies were the most common primary funder of Australian-led acute care trials (26%) and ACTA network acute care trials (74%), and acute care trials funded by government bodies were significantly more likely to propose and publish a health economic evaluation than acute care trials funded by other sources. The proportion of completed ACTA network acute care trials with a proposed health economic evaluation was more than three times higher than all acute care trials (53% versus 16%). Investigator-initiated clinical trials endorsed or run by clinical trial networks play a critical role in addressing clinically relevant research questions, influencing practice guidelines, identifying interventions or models of care that improve safety and quality, and determining practical strategies for more efficient resource use [6]. Continuing to invest in clinical trial networks that support funding for a health economist or a health economic evaluation may be particularly helpful in leveraging existing capacity in health economic evaluations and increasing the uptake of health economic evaluations alongside trials.

The results of health economic evaluations are becoming increasingly important as budgets and resources are limited, and priorities shift to respond to pressing health and social care problems. Both publicly and privately funded health systems, as well as national policy-making bodies and local-level funders, must justify investment in particular interventions, technologies, or programs over others [31, 32]. Similarly, health system decision-makers, policymakers, and funders benefit from information that substantiates disinvestment in interventions that are expensive but do not realize adequate health gains. Health economic evaluations alongside clinical trials are important for improving initial adoption decisions, examining the cost-effectiveness of interventions, addressing the requirements of regulatory bodies, and affecting reimbursement decisions for new medical technologies [9]. Although health economic evaluations form an essential component of health technology assessments, such as in the UK [33], Canada [34], and Australia [35], these assessments are often modeled assessments based on trials conducted elsewhere. Model-based economic evaluations use data from registries or the literature instead of patient-level data to estimate costs or effectiveness. Health economic evaluations alongside clinical trials allows for consideration of both the economic value and efficacy of therapies in the local context by accessing individual-level data. This allows for less biased, more robust estimates to be made [36] and, therefore, appears most likely to drive policymaking. Enhancing the capacity and willingness of clinical trialists to integrate health economic evaluations alongside clinical trials, therefore, can ensure the investment in and implementation of evidence-based interventions in the local context that maximize value, i.e., reduce or minimize costs and improve or maintain health outcomes.

### Strengths and limitations

This observational study utilized a systematic search of two clinical trial registries, as well as a survey of clinical trial networks, to capture all Australian-led clinical trials. Dual data types allowed us to corroborate one data type with the other and ensure all relevant trials were included in the analysis. We also validated our findings by conducting a subgroup analysis of completed acute care trials, which showed similar results to the analysis of all Australian-led trials. We searched for published health economic evaluations of acute care trials across Google, as well as academic databases, because some trials funded by government agencies do not have to publish results in the academic literature. We also published open-source code to improve the replicability of results and the application of these methods to other research questions ([13]; Additional file 2).

Synthesizing data across two registries did pose some challenges. Firstly, trials listed as phase 2/3 in the ANZCTR were included in the analysis despite phase 2 studies not typically including a health economic evaluation. Including phase 2/3 meant we may have included trials that were not fit for a health economic evaluation (e.g., small sample size or wrong outcomes). However, phase 3/4 and phase 4 trials had a similar proportion (6–8%) of trials with a health economic evaluation compared to phase 2/3 (8%), so we do not believe this substantively skewed our results. Second, trial registrations were systematically searched for economic-related keywords. It is possible that the registration did not state any economic-related outcomes or methods, but an economic evaluation was planned. It is also possible that the registration stated an economic-related outcome in the brief summary with no intention of conducting an economic analysis. However, we validated the findings by conducting a subgroup analysis of acute care trials, which had similar results to the analysis of all trials. Lastly, the analysis relied on trial sponsors accurately reporting and regularly updating the registration. For example, it is possible that many phase N/a trials were pilot or feasibility trials, but excluding these would have excluded all non-drug trials. We, therefore, believe that the approach taken for this observational study was robust and valid.

## Conclusions

Health economic evaluations alongside clinical trials inform resource allocation decisions and ensure clinical practice reflects what is most effective and efficient. Current uptake of health economic evaluations alongside Australian-led clinical trials, however, is low (11%), even among phase 3 trials. There is significant opportunity to embed health economic evaluations alongside Australian-led clinical trials. Doing so may further improve and accelerate the implementation of trial results into practice, particularly in areas of healthcare with high expenditure (e.g., acute care). Clinical trials endorsed or run by Australian clinical trial networks have higher uptake of health economic evaluations, so continuing to invest in clinical trial networks that support funding for a health economist or a health economic evaluation may be an effective strategy to build local health economic evaluation capacity and increase the uptake of health economic evaluations alongside trials.

## Supplementary Information

Additional file 1.

Additional file 2.

Additional file 3.

Additional file 4.

Additional file 5.

## Data Availability

The datasets generated and/or analyzed during the current study are available in the Australian New Zealand Clinical Trials Registry (ANZCTR) and ClinicalTrials.gov registries.

## Abbreviations

ACTA: Australian Clinical Trials Alliance
ANZCTR: Australian New Zealand Clinical Trials Registry
