# Supplementary material for: Uptake of health economic evaluations alongside clinical trials in Australia: an observational study
Source: Trials. 2024 Oct 22;25:705. doi: 10.1186/s13063-024-08562-3 (PMC11494774; doi:10.1186/s13063-024-08562-3)
Supplement: Supplementary file 1 — Additional file 1. [file 13063_2024_8562_MOESM1_ESM.pdf]

Additional File 1: STROBE Statement

	Item No	Recommendation	Page No
**Title and abstract**	1	(*a*) Indicate the study's design with a commonly used term in the title or the abstract	Page 1-3
		(*b*) Provide in the abstract an informative and balanced summary of what was done and what was found	Page 2-3
**Introduction**			
Background/rationale	2	Explain the scientific background and rationale for the investigation being reported	Page 3-4
Objectives	3	State specific objectives, including any prespecified hypotheses	Page 4
**Methods**			
Study design	4	Present key elements of study design early in the paper	Page 4-5
Setting	5	Describe the setting, locations, and relevant dates, including periods of recruitment, exposure, follow-up, and data collection	Page 4-5
Participants	6	(*a*) Give the eligibility criteria, and the sources and methods of selection of participants. Describe methods of follow-up	Not applicable
		(*b*) For matched studies, give matching criteria and number of exposed and unexposed	Not applicable
Variables	7	Clearly define all outcomes, exposures, predictors, potential confounders, and effect modifiers. Give diagnostic criteria, if applicable	Page 5-8
Data sources/ measurement	8*	For each variable of interest, give sources of data and details of methods of assessment (measurement). Describe comparability of assessment methods if there is more than one group	Page 4-5
Bias	9	Describe any efforts to address potential sources of bias	Not applicable
Study size	10	Explain how the study size was arrived at	Not applicable
Quantitative variables	11	Explain how quantitative variables were handled in the analyses. If applicable, describe which groupings were chosen and why	Page 5-8
Statistical methods	12	(*a*) Describe all statistical methods, including those used to control for confounding	Page 5-8
		(*b*) Describe any methods used to examine subgroups and interactions	Page 7-8
		(*c*) Explain how missing data were addressed	Not applicable
		(*d*) If applicable, explain how loss to follow-up was addressed	Not applicable
		(*e*) Describe any sensitivity analyses	Not applicable
**Results**			
Participants	13*	(a) Report numbers of individuals at each stage of study—eg numbers potentially eligible, examined for eligibility, confirmed eligible, included in the study, completing follow-up, and analysed	Not applicable
		(b) Give reasons for non-participation at each stage	Not applicable
		(c) Consider use of a flow diagram	Figure 1
Descriptive data	14*	(a) Give characteristics of study participants (eg demographic, clinical, social) and information on exposures and potential confounders	Not applicable
		(b) Indicate number of participants with missing data for each variable of interest	Not applicable

		(c) Summarise follow-up time (eg, average and total amount)	Not applicable
Outcome data	15*	Report numbers of outcome events or summary measures over time	Not applicable
Main results	16	(*a*) Give unadjusted estimates and, if applicable, confounder-adjusted estimates and their precision (eg, 95% confidence interval). Make clear which confounders were adjusted for and why they were included	Not applicable
		(*b*) Report category boundaries when continuous variables were categorized	Not applicable
		(*c*) If relevant, consider translating estimates of relative risk into absolute risk for a meaningful time period	Not applicable
Other analyses	17	Report other analyses done—eg analyses of subgroups and interactions, and sensitivity analyses	Page 9-10
**Discussion**			
Key results	18	Summarise key results with reference to study objectives	Page 11
Limitations	19	Discuss limitations of the study, taking into account sources of potential bias or imprecision. Discuss both direction and magnitude of any potential bias	Page 14-15
Interpretation	20	Give a cautious overall interpretation of results considering objectives, limitations, multiplicity of analyses, results from similar studies, and other relevant evidence	Page 11-14
Generalisability	21	Discuss the generalisability (external validity) of the study results	Page 12-14
**Other information**			
Funding	22	Give the source of funding and the role of the funders for the present study and, if applicable, for the original study on which the present article is based	Page 17
