# Supplementary material for: Uptake of health economic evaluations alongside clinical trials in Australia: an observational study
Source: Trials. 2024 Oct 22;25:705. doi: 10.1186/s13063-024-08562-3 (PMC11494774; doi:10.1186/s13063-024-08562-3)
Supplement: Supplementary file 2 — Additional file 2. [file 13063_2024_8562_MOESM2_ESM.pdf]

Additional File 2: R code used to filter trial registrations

```
## Purpose: Identifying Australian-led clinical trials and trials with a health economic evaluation
## Authors: Alayna Carrandi, Ava Tan, Sherrie Liu, Angie Barba, and Anais Charles-Nelson
## Date: 15 November 2023
## Recommended citation: Carrandi, A., Tan, A., Liu, S., Barba, A., & Charles-Nelson, A. (2023, November 22). Health economic evaluations alongside Australian-led clinical trials. Retrieved from osf.io/wq58t

## Start up -----

### Clear the environment
rm(list=ls())

### Load packages
### NB: you may need to install packages before loading
library('tidyverse')
library('here')
library('purrr')
library('janitor')
library('stringr')
library('dplyr')
library(readxl)
library(tidyr)
```

```
library(lubridate)

### Set path
### Replace the path below as needed:
# setwd("[insert path here]")

# SEARCH 1: Australian New Zealand Clinical Trials Registry (ANZCTR) ---------------------
----------------------------------------

## PART 1: Read data -----

### Download data from the ANZCTR website
#### ANZCTR public website --> search page: https://www.anzctr.org.au/TrialSearch.aspx
#### Filters:
##### 'Registry:' ANZCTR
##### 'Registration date:' any can be entered, to suit the timing of your research project
##### 'Countries of recruitment:' Australia
#### Click the blue search button, and then the blue 'Download button' (with the 'Download
ALL ANZCTR trials to Excel' option)

### Once data has been downloaded from the ANZCTR, please unzip the excel file, and put a
copy into the 'data' folder.
### Replace the file name below, as needed:
ANZCTRdataDownload <- "TrialDetails_20230616.xlsx"
```

```r
### Import this data into R.

#### One table is created for each sheet.

### Function: read_excel_allsheets(filename, single_frame = TRUE)

#### Original: https://rdrr.io/github/nick-moffitt/orderlabel/man/read_excel_allsheets.html

#### Modification: the title of each sheet originally had spaces. For the table names, I have
replaced the spaces with underscores ("_") - for the purpose of easy calling later on.

read_excel_allsheets <- function(filename, single_frame = TRUE) {
  sheets <- readxl::excel_sheets(filename)
  ldf <- lapply(sheets, function(X) readxl::read_excel(filename, sheet = X))
  names(ldf) <- make_clean_names(sheets)
  if(single_frame == TRUE){
    ldf <- ldf %>% dplyr::bind_rows(.id = 'sheet_name') %>% tibble::as_tibble()
  } else{
    list2env(ldf, envir = .GlobalEnv)
  }
  return(ldf)
}


### Using the function above to read the actual data

#### This take some time to run, don't worry.

read_excel_allsheets(
  here(ANZCTRdataDownload),
  FALSE)


# PART 2: Individual search rules -----
```

Apply search filters, and extract individual lists of unique trial ID's from each rule

Rule 1. Australian-led trials, i.e. trials where the institution of the lead investigator or the sponsor lead is in Australia

1.a Primary sponsor - country = "Australia" only, OR

1.b Principal investigator - country = "Australia" only

- ignore secondary sponsors

- ignore other collaborators

- ignore contact person for public queries

- ignore contact person for scientific queries

Caveat 1.a - primary sponsor - country = "Australia" only

```
#### colnames(trial)
subset1a <- trial %>%
  select(`TRIAL ID`, `PRIMARY SPONSOR COUNTRY`) %>%
  subset(`PRIMARY SPONSOR COUNTRY` == "Australia")
```

Caveat 1.b - principal investigator - country = "Australia" only

```
#### colnames(contacts)
subset1b <- contacts %>%
  select(`TRIAL ID`, `TYPE`, `COUNTRY`) %>%
  subset(`TYPE` == "Principal Investigator"
         & `COUNTRY`== "Australia")
```

Final list of trials that satisfy either 1.a OR 1.b

```
rule1output <- merge(x=subset1a, y=subset1b, by="TRIAL ID", all=TRUE)


### Rule 2. All randomised controlled intervention trials, any recruitment status
### Caveat 2.a - study type = "Interventional" (exclude "Observational")
### Caveat 2.b - comparator/control = EXCLUDE "Uncontrolled" (include all types of controls, including historical controls)
### Caveat 2.c - allocation to intervention = "Randomised controlled trial" (exclude "Non-randomised trial")
rule2output <- trial %>%
  select(`TRIAL ID`,`STUDY TYPE`,`CONTROL`,`ALLOCATION`) %>%
  subset(`STUDY TYPE` == "Interventional"
         & `CONTROL` != "Uncontrolled"
         & `ALLOCATION` == "Randomised controlled trial")


### Rule 3. Exclude phase 0, phase 1 and phase 2 trials
#### 'Phase' = EXCLUDE "Phase 0", "Phase 1", "Phase 1/Phase 2", "Phase 2"
#### Continue to include "Phase 2/Phase 3"
#### NB only drug trials will have explicit "Phase X" labels. Non-drug trials will not have these labels, and will NOT be filtered out.
rule3output <- trial %>%
  select(`TRIAL ID`,`PHASE`) %>%
  subset(`PHASE` != "Phase 0"
         & `PHASE` != "Phase 1"
         & `PHASE` != "Phase 1 / Phase 2"
         & `PHASE` != "Phase 2")
```

```
#Trials that fulfill ~all~ criteria -----
activeRules = list(rule1output,
                   rule2output,
                   rule3output)
ruleAllOutput_id <- activeRules %>%
  reduce(inner_join, by='TRIAL ID') %>%
  select('TRIAL ID')

# PART 3: Collapsing data tables -----
### Ensuring each trial exists in only 1 row, by merging repeater field values into 1 data item per trial.
### Distinguished by "*[descriptor of next value in a child hierarchy]*", "**[descriptor of next value in a sibling hierarchy]**"

### Secondary outcomes -----
#### Trials can have up to 40 multiple secondary outcomes.
#### In the raw secondary outcomes table, each secondary outcome exists in its own row.
secondary_outcome_collapsed <- secondary_outcome %>%
  unite(secoutcomeANDtime, OUTCOME:TIMEPOINT, sep= " *AT TIMEPOINT* ") %>%
  group_by(`TRIAL ID`) %>%
  summarise(secoutcomeANDtime = paste(secoutcomeANDtime, collapse = " **ANOTHER 2NDARY OUTCOME** "))

### Primary outcomes -----
```

```
primary_outcome_collapsed <- primary_outcome %>%
  unite(prioutcomeANDtime, OUTCOME:TIMEPOINT, sep= " *AT TIMEPOINT* ") %>%
  group_by(`TRIAL ID`) %>%
  summarise(prioutcomeANDtime = paste(prioutcomeANDtime, collapse = " **ANOTHER
PRIMARY OUTCOME** "))

### Secondary ID -----
secondary_id_collapsed <- secondary_id %>%
  group_by(`TRIAL ID`) %>%
  summarise(secID = paste(`SECONDARY ID`, collapse = " **ANOTHER ID** "))

### Condition code -----
condition_code_collapsed <- condition_code %>%
  unite(condcode, `CONDITION CATEGORY`:`CONDITION CODE`, sep= " *WITH
CODE* ") %>%
  group_by(`TRIAL ID`) %>%
  summarise(condcode = paste(condcode, collapse = " **ANOTHER CATEGORY** "))

### Intervention code -----
intervention_code_collapsed <- intervention_code %>%
  group_by(`TRIAL ID`) %>%
  summarise(intvcode = paste(`INTERVENTION CODE`, collapse = " **ANOTHER
CODE** "))

### Health condition -----
```

```
health_condition_collapsed <- health_condition %>%
  group_by(`TRIAL ID`) %>%
  summarise(healthcond = paste(`HEALTH CONDITION`, collapse = " **ANOTHER CONDITION** "))

## Hospital -----
hospital_collapsed <- hospital %>%
  group_by(`TRIAL ID`) %>%
  summarise(site = paste(`HOSPITAL`, collapse = " **ANOTHER SITE** "))

## Funding source -----
funding_source_collapsed <- funding_source %>%
  group_by(`TRIAL ID`) %>%
  summarise(funding_type = paste(`FUNDING SOURCE TYPE`, collapse = " **ANOTHER FUNDING TYPE** "))

## Summary results -----

rename(summary_results, journal_published = "RESULTS PUBLISHED IN JOURNAL?",
       else_published = "RESULTS PUBLISHED ELSEWHERE?",
       results_attachment = "RESULTS ATTACHMENT",
       results_summary = "RESULTS SUMMARY")

### The other tables have been ignored for now, as they are currently not relevant.
```

```
#### In the future, if any of those tables might be useful, they can be easily included by following the pattern of the code above.
#### The ignored tables currently are:
##### contacts
##### country_outside_australia
##### data_sharing_statement
##### ethics_committee
##### other_collaborator
##### postcode
##### secondary_sponsor

## Gather the relevant groups of data for those trials
### NB some data has been excluded for efficiency. For a list of dropped items, please see [PART 1 > Collapsing data tables]
#### It is very possible to include the data, by adding to: (i) [Part 1 > Collapsing data tables], and then (ii) the "list" function below.
ANZCTR <- list(ruleAllOutput_id,
               trial,
               secondary_id_collapsed,
               condition_code_collapsed,
               intervention_code_collapsed,
               primary_outcome_collapsed,
               secondary_outcome_collapsed,
               health_condition_collapsed,
               hospital_collapsed,
```

```
                              funding_source_collapsed,
                              summary_results) %>%
  reduce(left_join, by = "TRIAL ID")

## Save file as CSV to screen 'secondary_id_collapsed' for ClinicalTrials.gov IDs (begins
with NCT).
## Create a new CSV file with 'ACTRN' code in one column and 'NCT' code in another
column. This will be used to merge data files later.
write.csv(ANZCTR, "ANZCTR.csv", row.names=FALSE)

### Optional: Save file to avoid having to rerun the above code
### save(ANZCTR, file=[replace file name here]\\File Name)

# SEARCH 2: ClinicalTrials.gov (CTgov) ----------------------------------------------------------
--

## PART 1: Read data -----

### Download data from the CTgov website
#### CTgov public website --> search page: https://clinicaltrials.gov/
#### Filters:
##### 'Registration date:' since 2005 (ANZCTR inception)
##### 'Location:' Australia
#### Click the blue search button, and then the Download button
#### Select file format = CSV, results to download = All, data fields = select all
```

```
### Once data has been downloaded from the CTgov, put a copy into the 'data' folder.
### NB: Trials were then manually screened by one reviewer to confirm location of sponsor and investigator = Australia.
#### If the location of the lead investigator’s institution or sponsor lead was not in Australia, trials were deleted from CSV.
### Replace the file name below, as needed:
CTGOV <- read_csv("ctg-studies.csv",show_col_types = FALSE)


# PART 2: Individual search rules -----
## Apply search filters, and extract individual lists of unique trial ID's from each rule


## Identify randomized trials
CTGOV$study_design <- ifelse(grepl("Allocation: RANDOMIZED", CTGOV$'Study Design'), "Randomized", "Non-randomized")


## Identify purpose
CTGOV$primary_purpose <- ifelse(grepl("Primary Purpose: TREATMENT", CTGOV$'Study Design'), "Treatment",
                              ifelse(grepl("Primary Purpose: PREVENTION", CTGOV$'Study Design'), "Prevention",
                              ifelse(grepl("Primary Purpose: Diagnostic", CTGOV$'Study Design'), "Diagnostic",
                              ifelse(grepl("Primary Purpose: Supportive Care", CTGOV$'Study Design'), "Supportive Care",
```

```
                    ifelse(grepl("Primary Purpose: Screening", CTGOV$'Study Design'),
"Screening",
                    ifelse(grepl("Primary Purpose: Health Services Research",
CTGOV$'Study Design'), "Health Services Research",
                    ifelse(grepl("Primary Purpose: Basic Science", CTGOV$'Study Design'),
"Basic Science",
                    ifelse(grepl("Primary Purpose: Device Feasibility", CTGOV$'Study
Design'), "Device Feasibility",
                    ifelse(grepl("Primary Purpose: Other", CTGOV$'Study Design'), "Other",
                    "."))))))))



## Rule 1. Randomized trials, exclude phase 1|2 trials
CTGOV <- CTGOV %>%
  subset(`Study Type` == "INTERVENTIONAL"
      & study_design == "Randomized"
      & Phases != "EARLY_PHASE1"
      & Phases != "PHASE1"
      & Phases != "PHASE1|PHASE2"
      & Phases != "PHASE2")

# MERGE ANZCTR AND CLINICALTRIALS.GOV DATAFILES ----------------------------
---------------------------------
```

```
## Upload file with ACTRN and corresponding NCT numbers *See last step of search 1
above for instructions*
### Replace the file name below, as needed:
# anzctr_with_nct <- read.csv("[replace file name here].csv")
anzctr_with_nct$ACTRN <- as.numeric(anzctr_with_nct$ACTRN)
anzctr_with_nct$ACTRN <- format(anzctr_with_nct$ACTRN, scientific = FALSE)

ANZCTR$ACTRN <- as.numeric(ANZCTR$ACTRN)
ANZCTR$ACTRN <- format(ANZCTR$ACTRN, scientific = FALSE)

ANZCTR_NCT <- merge(ANZCTR, anzctr_with_nct, by='ACTRN', all.x = TRUE)

CTGOV <- CTGOV %>%
  rename(NCT =`NCT Number`)

all_trials <- merge(ANZCTR_NCT, CTGOV, by = 'NCT', all=TRUE, sort=FALSE)

## Create a new column which contains values from ANZCTR if they exist, otherwise, it
takes the values from CTGOV
all_trials$Combined_title <- ifelse(is.na(all_trials$'STUDY TITLE'), all_trials$'Study Title',
all_trials$'STUDY TITLE')
all_trials$Combined_acronym <- ifelse(is.na(all_trials$'TRIAL ACRONYM'),
all_trials$Acronym, all_trials$'TRIAL ACRONYM')
all_trials$Combined_interventions <- ifelse(is.na(all_trials$'INTERVENTIONS'),
all_trials$Interventions, all_trials$'INTERVENTIONS')
```

```
all_trials$Combined_studytype <- ifelse(is.na(all_trials$'STUDY TYPE'), all_trials$'Study
Type', all_trials$'STUDY TYPE')
all_trials$Combined_purpose <- ifelse(is.na(all_trials$'PURPOSE'),
all_trials$primary_purpose, all_trials$'PURPOSE')
all_trials$Combined_phase <- ifelse(is.na(all_trials$'PHASE'), all_trials$Phases,
all_trials$'PHASE')
all_trials$Combined_trialstatus <- ifelse(is.na(all_trials$'RECRUITMENT STATUS'),
all_trials$'Study Status', all_trials$'RECRUITMENT STATUS')
all_trials$Combined_summary <- ifelse(is.na(all_trials$'BRIEF SUMMARY'),
all_trials$'Brief Summary', all_trials$'BRIEF SUMMARY')
all_trials$Combined_condcode <- ifelse(is.na(all_trials$condcode), all_trials$Condition,
all_trials$condcode)
all_trials$Combined_prioutcome <- ifelse(is.na(all_trials$prioutcomeANDtime),
all_trials$'Primary Outcome Measures', all_trials$prioutcomeANDtime)
all_trials$Combined_secoutcome <- ifelse(is.na(all_trials$secoutcomeANDtime),
all_trials$'Secondary Outcome Measures', all_trials$secoutcomeANDtime)

all_trials$start_date_formatted <- as.Date(all_trials$'ANTICIPATED START DATE',format
= "%y")
all_trials$start_date_formatted_ctgov <- as.Date(all_trials$'Start Date',format = "%y")
all_trials$Combined_startdate <- ifelse(is.na(all_trials$start_date_formatted),
all_trials$start_date_formatted_ctgov, all_trials$start_date_formatted)
all_trials$Combined_startdate <- as.Date(all_trials$Combined_startdate)

# DATA CLEANING ----------------------------------------------------------------
```

```
## PART 1: Therapeutic area -----

all_trials$Alt_med <- ifelse(grepl("Alternative and complementary medicine",
all_trials$Combined_condcode), TRUE, FALSE)
all_trials$Anaesthetics <- ifelse(grepl("Anaesthesiology|Anesthesia",
all_trials$Combined_condcode), TRUE, FALSE)
all_trials$Blood <- ifelse(grepl("Blood|Deep Vein|Blood Coagulation
Disorders|Anaemia|Neutropenia", all_trials$Combined_condcode), TRUE, FALSE)
all_trials$Cancer <- ifelse(grepl("Cancer|Chemotherapy|Neoplasms|Radiotherapy|Colonic
Adenomas|Esophageal Stenosis|Febrile
Neutropenia|Maligna|Leukemia|Carcinoma|Melanoma|Tumor|Polyps|Polyp",
all_trials$Combined_condcode), TRUE, FALSE)
all_trials$Cardiovascular <- ifelse(grepl("Cardiovascular|Myocardial|Coronary
Artery|STEMI|Heart|Central Sympathetic Nervous|Endothelial
Dysfunction|Vascular|Arteriosclerosis|Cardiac|Arterial|Aortic|Hypertension|High Blood
Pressure|Ischemia|Hypercholesterolemia", all_trials$Combined_condcode), TRUE, FALSE)
all_trials$Diet_nutrition <- ifelse(grepl("Diet and
nutrition|Obesity|Hypercholesterolaemia|Overweight|Food|Cholesterol|Eating|Diet|Anorexia
Nervosa|Nutrition", all_trials$Combined_condcode), TRUE, FALSE)
all_trials$Ear <- ifelse(grepl("Ear|Hearing|Otitis Media", all_trials$Combined_condcode),
TRUE, FALSE)
all_trials$Emergency_med <- ifelse(grepl("Emergency medicine|Trauma|Traumatic Brain
Injury|Cardiac Arrest", all_trials$Combined_condcode), TRUE, FALSE)
```

```
all_trials$Eye <- ifelse(grepl("Eye|Diabetic macular|Myopia|Vision|Chorioretinopathy|Retinopathy|Presbyopia|Glaucoma|Meibomian Gland Dysfunction|Optometry|Contact Lens|macular degeneration", all_trials$Combined_condcode), TRUE, FALSE)
all_trials$Infection <- ifelse(grepl("Infection|HIV|Bacteria|Bacterial|Antimicrobial|Invasive Aspergillosis|Hypersensitivity Response|Mycobacteria|Immune Response|Tuberculosis|Head lice|Bronchiectasis|Cellulitis|Staphylococcus|Infections|Bronchiolitis|Otitis Media|Sepsis|Gastroenteritis|Shock", all_trials$Combined_condcode), TRUE, FALSE)
all_trials$Inflammatory <- ifelse(grepl("Inflammatory|Urticaria|Angioedema|Allergy|Inflammation|Chronic Rhinosinusitis|Vulvovaginal Atrophy|Allergic rhinitis|Eczema|Appendicitis|Glomerulonephritis|Ulcerative Colitis|Liver Disease|Allergy|Hepatitis", all_trials$Combined_condcode), TRUE, FALSE)
all_trials$Injury <- ifelse(grepl("Injuries|Mallet Finger|Accidental Falls|Articular Cartilage Defect|Falls|Cervical Injury|Fracture|Fractures|Burns", all_trials$Combined_condcode), TRUE, FALSE)
all_trials$Genetics <- ifelse(grepl("Human genetics|Cystic Fibrosis|Genetic|Epidermolysis Bullosa|Parkinson|Dravet Syndrome|Prader-Willi Syndrome|Hereditary Haemochromatosis|Familial", all_trials$Combined_condcode), TRUE, FALSE)
all_trials$Mental_health <- ifelse(grepl("Mental health|Schizophrenia|Antisocial|Psychotic|Phobia|Schizoaffective|Cannabis Use|Suicide|Psychoses|Insomnia|Substance|Drug and Alcohol|Tobacco Use Disorder|Drug Abuse|Methamphetamine Use Disorder|Depression|Bipolar|Depressive|Autistic Disorder|Anxiety|Impulsive Behavior|Alcohol Dependence|Loneliness|Compulsive|Alcohol Use", all_trials$Combined_condcode), TRUE, FALSE)
```

```
all_trials$Metabolic <- ifelse(grepl("Metabolic|Diabetes|Endocrine|Hyperglycemia|Diabetic
Ketoacidosis|Pancreatic|Insulinemia|Glycaemia|Metabolism|Insulin|Preeclampsia",
all_trials$Combined_condcode), TRUE, FALSE)
all_trials$Musculoskeletal <- ifelse(grepl("Musculoskeletal|Chronic Groin Pain|Basilar
Artery Occlusion|Articular Cartilage
Defect|Dystrophy|Osteoarthritis|Spondylolisthesis|Supraspinatus Tear|Joint
Disease|Pain|Arthritis|Hernia", all_trials$Combined_condcode), TRUE, FALSE)
all_trials$Nuerological <- ifelse(grepl("Nuerological|Migraine|Parkinson's|Sclerosis|Cerebral
Palsy|Wernicke-Korsakoff|Seizure|Lennox Gastaut Syndrome|Dementia|Amnesia|Cognition
Disorders|Neurocognitive Impairment|Delirium|Cognitive Impairment|Brain|Effect on
Learning|Cognitive Decline|Insomnia|Sleep", all_trials$Combined_condcode), TRUE,
FALSE)
all_trials$Gastro <- ifelse(grepl("Oral and gastrointestinal|Gastrointestinal|Microbial
Colonization|Chrohn's|Ulcerative Colitis|Peritoneal|Irritable
Bowel|Gastroenteritis|Chron|Colorectal Disorders", all_trials$Combined_condcode), TRUE,
FALSE)
all_trials$Physio <- ifelse(grepl("Physical medicine|Rehabilitation|Physical Therapy",
all_trials$Combined_condcode), TRUE, FALSE)
all_trials$Renal <- ifelse(grepl("Renal|Urogenital|Diabetic Nephropathy|Diabetic
Nephropathies|Hypovolemia|Urinary|FSGS|Kidney|Proteinuria|Haemochromatosis|Calciphyl
axis", all_trials$Combined_condcode), TRUE, FALSE)
all_trials$Public_health <- ifelse(grepl("Public health|Knowledge|Healthy|Help-
Seeking|Wellness|Behaviour|Behaviours|Behaviors|Independent
Living|Wellbeing|Learning|Education", all_trials$Combined_condcode), TRUE, FALSE)
```

```
all_trials$Reproductive <- ifelse(grepl("Reproductive
health|Placenta|Postpartum|Pregnancy|Fetal|Menopausal|Libido Disorder|Erectile
Dysfunction|Preterm|Ovary|Perimenopausal|Menstrual|Infertility|Preeclampsia",
all_trials$Combined_condcode), TRUE, FALSE)
all_trials$Respiratory <-
ifelse(grepl("Respiratory|Asthma|Dyspnea|Breathing|Lung|Influenza|Apnea|Emphysema|Pul
monary|Bronchopulmonary Dysplasia|COPD|Covid19|Pneumonia|COVID-19|Fibrosis",
all_trials$Combined_condcode), TRUE, FALSE)
all_trials$Skin <- ifelse(grepl("Skin|Keloid|Pressure Ulcers|Impetigo|Epidermolysis
Bullosa|Atopic Dermatitis|Dermatitis|Eczema", all_trials$Combined_condcode), TRUE,
FALSE)
all_trials$Surgery <- ifelse(grepl("Surgery|Laparoscopic
cholecystectomy|Catheterization|Catheterisation|Colonoscopy|Intubation|Athroplasty|Laparos
copy|Surgical|Transplantation|Biopsy|Wound Complication",
all_trials$Combined_condcode), TRUE, FALSE)
all_trials$Stroke <- ifelse(grepl("Stroke|Hemorrhage|Hemorrhages|Intracranial
Aneurysms|Haemorrhage|Haemorrhagic", all_trials$Combined_condcode), TRUE, FALSE)
all_trials$Othercat <- ifelse(grepl("Other|Electonic Medical
Record|Videoconferencing|Orthodontic|Peri-Implantitis|Underbite",
all_trials$Combined_condcode), TRUE, FALSE)

## Identifying critical care trials
all_trials$Critical_care_title <- ifelse(grepl("Critical Illness|Mechanical
Ventilation|Shock|Sepsis|Intubation|Critically Ill|Intensive Care Unit|Community-acquired
```

```
Pneumonia|Extracorporeal Membrane Oxygenation", all_trials$Combined_title), TRUE,
FALSE)
all_trials$Critical_care_intervention <- ifelse(grepl("Critical Illness|Mechanical
Ventilation|Shock|Sepsis|Intubation|Critically Ill|Intensive Care Unit|Community-acquired
Pneumonia|Extracorporeal Membrane Oxygenation",all_trials$Combined_interventions),
TRUE, FALSE)
all_trials$Critical_care_cond <- ifelse(grepl("Critical Illness|Mechanical
Ventilation|Shock|Sepsis|Intubation|Critically Ill|Intensive Care Unit|Community-acquired
Pneumonia|Extracorporeal Membrane Oxygenation", all_trials$Combined_condcode),
TRUE, FALSE)
all_trials$Critical_care_healthcond <- ifelse(grepl("Critical Illness|Mechanical
Ventilation|Shock|Sepsis|Intubation|Critically Ill|Intensive Care Unit|Community-acquired
Pneumonia|Extracorporeal Membrane Oxygenation", all_trials$healthcond), TRUE, FALSE)

all_trials$Critical_care <- ifelse(all_trials$Critical_care_title == TRUE|
all_trials$Critical_care_scititle == TRUE| all_trials$Critical_care_intervention == TRUE|
all_trials$Critical_care_cond == TRUE| all_trials$Critical_care_healthcond == TRUE,
FALSE)

## PART 2: Phase categories -----

all_trials$Combined_phase <- ifelse(all_trials$Combined_phase == "PHASE2|PHASE3",
"Phase 2 / Phase 3", all_trials$Combined_phase)
all_trials$Combined_phase <- ifelse(all_trials$Combined_phase == "Not Applicable",
"N/A", all_trials$Combined_phase)
```

```
all_trials$Combined_phase <- ifelse(all_trials$Combined_phase == "PHASE3", "Phase 3",
all_trials$Combined_phase)
all_trials$Combined_phase <- ifelse(all_trials$Combined_phase == "PHASE4", "Phase 4",
all_trials$Combined_phase)

## PART 3: Trial status -----

all_trials$status_updated <- ifelse(all_trials$Combined_trialstatus == "COMPLETED" |
all_trials$Combined_trialstatus == "Completed", "Completed",
                          ifelse(all_trials$Combined_trialstatus == "Recruiting" |
all_trials$Combined_trialstatus == "RECRUITING" | all_trials$Combined_trialstatus ==
"ENROLLING_BY_INVITATION", "Recruiting",
                          ifelse(all_trials$Combined_trialstatus == "Active, not recruiting" |
all_trials$Combined_trialstatus == "Not yet recruiting" | all_trials$Combined_trialstatus ==
"NOT_YET_RECRUITING" | all_trials$Combined_trialstatus ==
"ACTIVE_NOT_RECRUITING", "Not started",
                          "Abandoned")))

# IDENTIFYING TRIALS WITH A HEALTH ECONOMIC EVALUATION ------------------
-------------------------------------------

all_trials$HEAT_title <- ifelse(grepl("economic|quality-adjusted|quality adjusted|cost-
effectiveness|cost effectiveness|cost-utility|cost utility|incremental net benefit|incremental
cost-effectiveness|cost per quality-adjusted|cost per quality adjusted|cost per
qaly|cost/qaly|cost per life year|cost per LY|cost per LYS|cost/LYS|cost per life saved|cost per
```

LS|cost/LS|cost benefit|cost-benefit|cost minimisation|cost minimization|cost-minimisation|cost-minimization", all_trials$Combined_title), TRUE, FALSE)

all_trials$HEAT_intervention <- ifelse(grepl("economic|quality-adjusted|quality adjusted|cost-effectiveness|cost effectiveness|cost-utility|cost utility|incremental net benefit|incremental cost-effectiveness|cost per quality-adjusted|cost per quality adjusted|cost per qaly|cost/qaly|cost per life year|cost per LY|cost per LYS|cost/LYS|cost per life saved|cost per LS|cost/LS|cost benefit|cost-benefit|cost minimisation|cost minimization|cost-minimisation|cost-minimization", all_trials$Combined_interventions), TRUE, FALSE)

all_trials$HEAT_primary <- ifelse(grepl("economic|quality-adjusted|quality adjusted|cost-effectiveness|cost effectiveness|cost-utility|cost utility|incremental net benefit|incremental cost-effectiveness|cost per quality-adjusted|cost per quality adjusted|cost per qaly|cost/qaly|cost per life year|cost per LY|cost per LYS|cost/LYS|cost per life saved|cost per LS|cost/LS|cost benefit|cost-benefit|cost minimisation|cost minimization|cost-minimisation|cost-minimization", all_trials$Combined_prioutcome), TRUE, FALSE)

all_trials$HEAT_secondary <- ifelse(grepl("economic|quality-adjusted|quality adjusted|cost-effectiveness|cost effectiveness|cost-utility|cost utility|incremental net benefit|incremental cost-effectiveness|cost per quality-adjusted|cost per quality adjusted|cost per qaly|cost/qaly|cost per life year|cost per LY|cost per LYS|cost/LYS|cost per life saved|cost per LS|cost/LS|cost benefit|cost-benefit|cost minimisation|cost minimization|cost-minimisation|cost-minimization", all_trials$Combined_secoutcome), TRUE, FALSE)

all_trials$HEAT_stats <- ifelse(grepl("economic|quality-adjusted|quality adjusted|cost-effectiveness|cost effectiveness|cost-utility|cost utility|incremental net benefit|incremental cost-effectiveness|cost per quality-adjusted|cost per quality adjusted|cost per qaly|cost/qaly|cost per life year|cost per LY|cost per LYS|cost/LYS|cost per life saved|cost per

```
LS|cost/LS|cost benefit|cost-benefit|cost minimisation|cost minimization|cost-minimisation|cost-minimization", all_trials$`STATISTICAL METHODS`), TRUE, FALSE)
all_trials$HEAT_briefsummary <- ifelse(grepl("economic|quality-adjusted|quality adjusted|cost-effectiveness|cost effectiveness|cost-utility|cost utility|incremental net benefit|incremental cost-effectiveness|cost per quality-adjusted|cost per quality adjusted|cost per qaly|cost/qaly|cost per life year|cost per LY|cost per LYS|cost/LYS|cost per life saved|cost per LS|cost/LS|cost benefit|cost-benefit|cost minimisation|cost minimization|cost-minimisation|cost-minimization", all_trials$Combined_summary), TRUE, FALSE)
all_trials$HEAT_resultsummary <- ifelse(grepl("economic|quality-adjusted|quality adjusted|cost-effectiveness|cost effectiveness|cost-utility|cost utility|incremental net benefit|incremental cost-effectiveness|cost per quality-adjusted|cost per quality adjusted|cost per qaly|cost/qaly|cost per life year|cost per LY|cost per LYS|cost/LYS|cost per life saved|cost per LS|cost/LS|cost benefit|cost-benefit|cost minimisation|cost minimization|cost-minimisation|cost-minimization", all_trials$`RESULTS SUMMARY`), TRUE, FALSE)
all_trials$HEAT_dataanalysis <- ifelse(grepl("economic|quality-adjusted|quality adjusted|cost-effectiveness|cost effectiveness|cost-utility|cost utility|incremental net benefit|incremental cost-effectiveness|cost per quality-adjusted|cost per quality adjusted|cost per qaly|cost/qaly|cost per life year|cost per LY|cost per LYS|cost/LYS|cost per life saved|cost per LS|cost/LS|cost benefit|cost-benefit|cost minimisation|cost minimization|cost-minimisation|cost-minimization", all_trials$`DATA ANALYSIS`), TRUE, FALSE)

all_trials$HEAT <- ifelse(all_trials$HEAT_title == "TRUE" | all_trials$HEAT_intervention == "TRUE" | all_trials$HEAT_primary == "TRUE" | all_trials$HEAT_secondary == "TRUE" | all_trials$HEAT_stats == "TRUE" | all_trials$HEAT_briefsummary == "TRUE" |
```

```
all_trials$HEAT_resultsummary == "TRUE" | all_trials$HEAT_dataanalysis == "TRUE",
TRUE, FALSE)


### Optional: Save file to avoid having to rerun the above code
### save(all_trials, file=[Path to export the DataFrame]\\File Name)
```
