# Supplementary material for: Uptake of health economic evaluations alongside clinical trials in Australia: an observational study
Source: Trials. 2024 Oct 22;25:705. doi: 10.1186/s13063-024-08562-3 (PMC11494774; doi:10.1186/s13063-024-08562-3)
Supplement: Supplementary file 3 — Additional file 3. [file 13063_2024_8562_MOESM3_ESM.pdf]

Additional File 3: Extraction form used for all completed acute care trials

Trial Name
Australian and New Zealand Clinical Trial Registry ID
Clinicaltrials.gov ID
Endorsed or run by Australian Clinical Trails Alliance (ACTA) network?
Trial Phase
Start date year
Funding source
Primary sponsor type
Purpose
Comparator
Control
Endpoint
Publication reference for main publication
Significant/non-significant results for primary outcome
Was the protocol and/or statistical analysis plan published?
Was a health economic evaluation planned?
Was a health economic evaluation included in the budget?
Was a health economic evaluation published?
Health economic evaluation publication reference. *If the health economic evaluation results were published within the primary publication, put 'within primary publication'.*
Economic methodology
Primary publication year
Health economic evaluation publication year

Time between primary and economic evaluation
Has the implementation of these results in clinical practice been evaluated? *If available and known, indicate where these results can be found (e.g., primary publication, other reference).*
Are there any other publications related to this trial? *If so, provide a link or reference.*
