# Supplementary material for: Uptake of health economic evaluations alongside clinical trials in Australia: an observational study
Source: Trials. 2024 Oct 22;25:705. doi: 10.1186/s13063-024-08562-3 (PMC11494774; doi:10.1186/s13063-024-08562-3)
Supplement: Supplementary file 4 — Additional file 4. [file 13063_2024_8562_MOESM4_ESM.pdf]

Additional File 4: Characteristics of Australian-led interventional randomized clinical trials

**Descriptive characteristic**	**All trials**		**ACTA network trials**		**Trials with a health economic evaluation**		**Trials without a health economic evaluation**	
	n = 9251		n = 227		n = 1,034		n = 8217	
Therapeutic area	n	%	n	%	n	%	n	%
Anaesthetics	514	5.6	24	10.6	31	3.0	483	5.9
Blood	87	0.9	1	0.4	7	0.7	80	1.0
Cancer	861	9.3	97	42.7	139	13.4	722	8.8
Cardiovascular	932	10.1	2	0.9	97	9.4	835	10.2
Critical care	181	2.0	46	20.3	41	4.0	140	1.7
Diet and nutrition	935	10.1	3	1.3	77	7.5	858	10.4
Ear	55	0.6	0	0.0	9	0.9	46	0.6
Emergency medicine	88	1.0	17	7.5	28	2.7	60	0.7
Eye	182	2.0	13	5.7	9	0.9	173	2.1
Infection	405	4.4	4	1.8	48	4.6	357	4.3

Inflammatory and immune system	274	3.0	3	1.3	17	1.6	257	3.1
Injury and accidents	442	4.8	3	1.3	72	7.0	370	4.5
Human genetics and inherited disorders	158	1.7	0	0.0	11	1.1	147	1.8
Mental health	1017	11.0	8	3.5	109	10.5	908	11.1
Metabolic and endocrine	769	8.3	2	0.9	65	6.3	704	8.6
Musculoskeletal	1156	12.5	14	6.2	114	11.0	1042	12.7
Neurological	356	3.9	6	2.6	41	4.0	315	3.8
Oral and gastrointestinal	655	7.1	23	10.1	54	5.2	601	7.3
Physical medicine / rehabilitation	720	7.8	2	0.9	103	10.0	617	7.5
Renal and urogenital	289	3.1	19	8.4	32	3.1	257	3.1
Public health	51	0.6	0	0.0	4	0.4	47	0.6
Reproductive health and childbirth	84	0.9	5	2.2	10	1.0	74	0.9

Respiratory	706	7.6	19	8.4	70	6.8	636	7.7
Skin	180	2.0	8	3.5	30	2.9	150	1.8
Surgery	533	5.8	10	4.4	52	5.0	481	5.9
Stroke	201	2.2	0	0.0	29	2.8	172	2.1
Other	3936	42.6	34	15.0	422	40.8	3514	42.8
Phase*	n	%	n	%	n	%	n	%
N/a (non-drug trials)	6949	75.1	31	13.7	806	78.0	6143	74.8
Phase 2/3	214	2.3	1	0.4	18	1.7	196	2.4
Phase 3	963	10.4	167	73.6	132	12.8	831	10.1
Phase 3/4	170	1.8	3	1.3	14	1.4	156	1.9
Phase 4	955	10.3	25	11.0	64	6.2	891	10.8
Status*	n	%	n	%	n	%	n	%
Active, not recruiting	495	5.4	13	5.7	99	9.6	396	4.8
Complete	3815	41.2	136	59.9	339	32.8	3476	42.3
Not yet recruiting	2025	21.9	2	0.9	211	20.4	1814	22.1
Recruiting	2131	23.0	71	31.3	316	30.6	1815	22.1

Abandoned[a]	697	7.5	5	2.2	66	6.4	631	7.7
Unknown status	88	1.0	0	0.0	3	0.3	85	1.0
Registration date	median	IQR	median	IQR	median	IQR	median	IQR
Year	2016	2013 - 2019	2016	2010 - 2019	2017	2014 - 2020	2014	2009 - 2019

Note: IQR = Interquartile range

[a]Status included stopped early, suspended, terminated, or withdrawn.

*Significant ($p < 0.05$) association with having a proposed health economic evaluation, using univariate analyses (Monte Carlo simulation)
