# Supplementary material for: Uptake of health economic evaluations alongside clinical trials in Australia: an observational study
Source: Trials. 2024 Oct 22;25:705. doi: 10.1186/s13063-024-08562-3 (PMC11494774; doi:10.1186/s13063-024-08562-3)
Supplement: Supplementary file 5 — Additional file 5. [file 13063_2024_8562_MOESM5_ESM.pdf]

Additional File 5: Characteristics of Australian-led completed acute care trials, including emergency medicine, critical care, surgery, and injury trials

**Descriptive characteristic**	**Total**		**ACTA network trials**		**Trials with a proposed health economic evaluation**		**Trials without a proposed health economic evaluation**	
	n = 324		n = 38		n = 52		n = 272	
Phase	n	%	n	%	n	%	n	%
Not applicable (non-drug trials)	222	68.5	7	18.4	32	61.5	190	69.9
2/3	8	2.5	1	2.6	2	3.9	6	2.2
3	39	12.0	21	55.3	14	26.9	25	9.2
3/4	6	1.9	1	2.6	0	0.0	6	2.2
4	49	15.1	8	21.1	4	7.7	45	16.5
Registration date	median	IQR	median	IQR	median	IQR	median	IQR
Year	2014	2010 - 2017	2012	2009 - 2016	2014	2011 - 2016	2014	2010 - 2017

Proposed health economic evaluation	n	%	n	%	n	%	n	%
Yes	52	16.1	20	52.6	-	-	-	-
No	272	84.0	18	47.4	-	-	-	-
Published health economic evaluation	n	%	n	%	n	%	n	%
Yes	14	4.3	8	21.1	14	26.9	-	-
No	13	4.0	1	2.6	13	25.0	-	-
Ongoing	25	7.7	11	29.0	25	48.1	-	-
No proposed health economic evaluation	272	84.0	18	47.4	-	-	-	-
Primary funding source*	n	%	n	%	n	%	n	%
Government body	84	25.9	28	73.7	33	63.5	51	18.8
Hospital	75	23.2	1	2.6	5	9.6	70	25.7
University	19	5.9	0	0.0	1	1.9	18	6.6
Commercial sector/industry	23	7.1	3	7.9	4	7.7	19	7.0

Charities/societies/foundations	50	15.4	5	13.2	5	9.6	45	16.5
Other collaborative groups	17	5.3	1	2.6	3	5.8	14	5.2
Self-funded/unfunded	48	14.8	0	0.0	1	1.9	47	17.3
Other	8	2.5	0	0.0	0	0.0	8	2.9
Primary sponsor type	n	%	n	%	n	%	n	%
Government body	5	1.5	1	2.6	1	1.9	4	1.5
Hospital	117	36.1	9	23.7	13	25.0	104	38.2
University	66	20.4	3	7.9	17	32.7	49	18.0
Commercial sector/industry	8	2.5	2	5.3	2	3.9	6	2.2
Charities/societies/foundations	10	3.1	4	10.5	2	3.9	8	2.9
Other collaborative groups	18	5.6	11	29.0	5	9.6	13	4.8
Individual	90	27.8	5	13.2	8	15.4	82	30.2
Other	7	2.2	1	2.6	1	1.9	6	2.2
None	3	0.9	2	5.3	0	0.0	3	1.1
Comparator/Control	n	%	n	%	n	%	n	%
Standard care	141	43.5	24	63.2	31	59.6	110	40.4

Wait-list control	8	2.5	0	0.0	0	0.0	8	2.9
Placebo	31	9.6	3	7.9	1	1.9	30	11.0
Cross-over trial	5	1.5	0	0.0	0	0.0	5	1.8
Other	139	42.9	11	29.0	20	38.5	119	43.8
Purpose	n	%	n	%	n	%	n	%
Diagnosis	7	2.2	1	2.6	1	1.9	6	2.2
Education	23	7.1	1	2.6	2	3.9	21	7.7
Prevention	77	23.8	4	10.5	15	28.9	62	22.8
Treatment	217	67.0	32	84.2	34	65.4	183	67.3
Endpoint	n	%	n	%				
Efficacy	245	75.6	25	65.8	38	73.1	207	76.1
Safety	11	3.4	2	5.3	1	1.9	10	3.7
Safety/efficacy	63	19.4	10	26.3	13	25.0	50	18.4
Other	5	1.5	1	2.6	0	0.0	5	1.8

Note: ACTA = Australian Clinical Trials Alliance; IQR = Interquartile range

*Significant ($p < 0.05$) independent association with proposing and publishing a health economic evaluation, accounting for phase, registration year, primary sponsor type, and comparator
